# Prevalence of occupational stress in health care personnel during the COVID-19 pandemic: a cross-sectional study

**DOI:** 10.47626/1679-4435-2022-957

**Published:** 2024-02-16

**Authors:** Kairo Silvestre Meneses Damasceno, Arthur Pinto Silva, Claudeone Vieira Santos, Janaina de Oliveira Castro, André da Silva dos-Santos, André Luiz Bispo Pereira, Rodrigo Fernandes Weyll Pimentel, Magno Conceição das Merces

**Affiliations:** 1 Departamento de Ciências da Vida, Universidade do Estado da Bahia, Salvador, BA, Brazil

**Keywords:** occupational stress, occupational health, primary health care, estresse ocupacional, saúde do trabalhador, atenção primária à saúde

## Abstract

**Introduction:**

Occupational stress has been exacerbated in health care personnel during the COVID-19 pandemic, which can harm the health of professionals, managers and the population.

**Objectives:**

To estimate the prevalence of occupational stress in professionals of the Family Health Strategy of a Health District in the city of Salvador, Bahia, Brazil.

**Methods:**

This is a cross-sectional study carried out with 105 professionals from the Family Health Strategy teams of three Family Health Units in a Health District in the city of Salvador, Bahia, Brazil. A questionnaire with sociodemographic and work information and the Work Stress Scale were applied. Numerical stress values were categorized, from the average, into low and high stress levels. Measures of central tendency and bivariate analysis between stress and other variables were calculated.

**Results:**

The high level of stress presented a prevalence of 46.7%, with the following most punctuated TSE stressors: lack of qualifications, few prospects for career growth, deficiency in the disclosure of decisions, discrimination at work and lack of autonomy.

**Conclusions:**

The prevalence of a high level of occupational stress among health professionals at Family Health Units reached 46.7% of the sample studied; a fact that deserves awareness on the part of managers regarding the promotion and protection of the health of health care personnel.

## Abstract (pt)

**Introdução:**

O estresse ocupacional tem sido exacerbado nos trabalhadores de saúde durante a pandemia da covid-19, podendo trazer prejuízos à saúde dos profissionais, aos gestores e à população.

**Objetivos:**

Estimar a prevalência do estresse ocupacional em profissionais da Estratégia Saúde da Família de um distrito sanitário do município de Salvador, Bahia, Brasil.

**Métodos:**

Trata-se de um estudo transversal realizado com 105 profissionais das equipes da Estratégia Saúde da Família de três Unidades de Saúde da Família de um distrito sanitário do município de Salvador, Bahia, Brasil. Foram aplicados um questionário com informações sociodemográficas e laborais e a Escala de Estresse no Trabalho. Os valores numéricos do estresse foram categorizados, a partir da média, em baixo e alto nível do estresse. Foram calculadas as medidas de tendência central e a análise bivariada entre o estresse e as demais variáveis.

**Resultados:**

O nível alto do estresse apresentou-se com uma prevalência de 46,7%, com os seguintes fatores estressores da Escala de Estresse no Trabalho mais pontuados: deficiência de capacitações, poucas perspectivas de crescimento na carreira, deficiência na divulgação das decisões, discriminação no trabalho e falta de autonomia.

**Conclusões:**

A prevalência do nível alto de estresse ocupacional entre os profissionais de saúde das Unidades de Saúde da Família alcançou 46,7% da amostra estudada; fato que merece sensibilização por parte dos gestores quanto à promoção e à proteção à saúde dos trabalhadores de saúde.

## INTRODUCTION

Occupational stress can be defined as physical and mental health changes in workers as a result of stressful factors in the workplace, such as excessive demands.^1^

Endocrinologist Hans Selye was the first scientist to study stress and its effects on the body defining it as the General Adaptation Syndrome (GAS) due to the body's reactions in an attempt to adapt to situations requiring effort. GAS was characterized by the alarm, resistance, and exhaustion phases.^2^

In the alarm phase, an immediate organic response to the stressor occurs, with an increase in heart rate, blood pressure, respiratory rate, anxiety, and pupil dilation. In the subsequent resistance phase, the body reacts to the continuation of the stressor with an increase in the cerebral cortex, gastric ulcers, insomnia, irritability, and a decrease in libido. If this progresses, the exhaustion phase is reached, with the same signs and symptoms as the alarm phase, physical and mental exhaustion, and failures in the functioning of the body^1,2^

The main stressors at workplace can be related to: the nature of the job, pressure for greater productivity, the need for new skills, multiple employment relationships, experiencing critical situations, routine, low pay, responsibilities, professional devaluation, lack of autonomy in decision-making, precarious working conditions, complex tasks, inadequate resources, interpersonal relationships, overload, working hours, among others.^3,4,5,6^

The COVID-19 pandemic has brought new elements that have exacerbated health care personnel's occupational stress, such as the fear of being fired and losing their livelihoods; the fear of being infected and placed in isolation, apart from their families; increased workload and long working hours; children at home due to the closure of schools; the need to keep up to date with the new disease; difficult decisions on therapeutic choices; mourning the losses of patients and colleagues; stigma generated in the population towards health care personnel who are in contact with COVID-19 carriers, among others.^7,8^

Occupational stress is an exposure factor for physical problems, such as musculoskeletal disorders, high blood pressure and other cardiovascular disorders, gastrointestinal problems, abdominal adiposity, metabolic syndrome and, eventually, incapacity for work, and death.^4,5,9^

As for emotional and cognitive symptoms of occupational stress, the main ones are: anxiety, anguish, anger, irritability, frustration, worry, depression, emotional hypersensitivity, tension, reduced attention and concentration, memory loss, aggressiveness, among others. Behavioral symptoms can also occur, such as increased alcohol and drug consumption, absenteeism, and sleep disorders.^3,10^

Furthermore, occupational stress can have an impact on organizations through costs associated with absenteeism, work delays and staff turnover, reduced performance and productivity, an increase in unsafe working practices, accident rates, and customer complaints; replacement of absent workers; training of replacement workers; among others. It is therefore a serious public health problem in modern society, which has consequences for employers, employees, and society as a whole.^3,10^

Given this context, this study aims to estimate the prevalence and associated factors of occupational stress in Family Health Strategy (FHS) personnel in a health district (HD) in Salvador, Bahia, Brazil, during the COVID-19 pandemic.

## METHODS

A cross-sectional study was conducted with FHS personnel in a HD in Salvador, Bahia, Brazil.

A cross-sectional study is an investigation on the exposure to an event or disease in a given population at a certain time. It is a method used to detect the prevalence of diseases and groups most affected.^11^

Districting means organizing health services and facilities into a network, according to geographical, population, epidemiological, management, and political criteria. It helps to ensure comprehensive care, inters ectorality, social participation, and effective services.^12^

The study was conducted in 3 Family Health Units (FHUs) in a HD in Salvador from March to May 2021. All health care personnel working in the family health teams were invited to be interviewed, totaling 13 teams and 145 workers, including registered nurses, certified nurses, dentists, dental assistants, community health care personnel, and physicians. The study inclusion criterion was being enrolled in a family health team. A total of 29 people refused, 6 were on medical leave, 2 were on maternity leave, and 3 were working remotely, totaling 105 participants.

Volunteer participants answered a sociodemographic and work-related questionnaire and the Work Stress Scale (WSS), a tool that provides diagnoses of stress in the workplace of organizations, guiding strategies aimed at the quality of life of workers.^13^

The WSS is a 23-item questionnaire which addresses a stressful stimulus and a reaction to it. It was validated and translated into Brazilian Portuguese by Paschoal & Tamayo.^13^ Cronbach’s alpha coefficient was 0.91. Each item is accompanied by Likert-type answers with the following alternatives: 1. Strongly Disagree; 2. Disagree; 3. Partly Agree; 4. Agree; and 5. Strongly Agree. The higher the sum, the greater the stress.^13^

Occupational stress therefore takes on a numerical score for each individual. After the interviews, each participant’s stress level was calculated (sum of the Likert divided by 23). These scores displayed normal distribution using the Kolmogorov-Smirnov test. The overall mean of the numerical stress scores (2.81) was used as a cut-off point to categorize stress as high or low level.

Pearson’s chi-squared test was used for the bivariate analysis, considering a p-value of less than 0.05 as statistically significant. The data were tabulated in MS Excel and Stata 11.0 was used for statistical analyses.

The Universidade do Estado da Bahia (UNEB, Bahia State University) Research Ethics Committee (REC) approved the study according to opinion 4.478.349. The human research guidelines of Resolution 466/2012 of the Conselho Nacional de Saúde (Brazil National Health Council) and the principles of the Declaration of Helsinki were observed. Those who agreed to participate in the study signed the Informed Consent Form (ICF).

## RESULTS

The study involved 105 health care personnel. Individuals up to 45 years old (60.95%), women (91.43%), of African descent (50.48%), community health care personnel (55.23%), undergraduate/graduate schooling (51.43%), up to 7 years in the FHS (54.29%), not working outside the FHU (79.05%), weekly workload up to 40 hours (81.90%), not on night duty (88.57%), with a family income of more than two Brazilian minimum wages (56.19%), economic discontent (82.86%), unmarried (56.19%), with 1 to 2 children (66.67%), a permanent job position (95.2%), and having already experienced some violence at the FHU (63.81%), as shown in Table 1.

**Table 1:** Sociodemographic and work-related characteristics of Family Health Strategy health professionals in a health district in Salvador, Bahia, Brazil, 2021

Characteristics	n	%
Age (years)		
<45	64	60.95
>45	41	39.05
Sex		
Male	9	8.57
Female	96	91.43
Skin color		
White	08	7.62
Brown	44	41.90
Black	53	50.48
Professional title		
Physician	7	6.67
Registered nurse	8	7.62
Dentist	8	7.62
Dental assistant	8	7.62
Certified nurse	16	15.24
Health community agent	58	55.23
Education		
Undergraduate/graduate	54	51.43
Elementary/high school	51	48.57
Occupation time at the FHU (years)		
<7	57	54.29
>7	48	45.71
Works outside the FHU		
No	83	79.05
Yes	22	20.95
Characteristics	n	%
Weekly working hours	86	81.90
<40	19	18.10
>40		
Night shift		
No	93	88.57
Yes	12	11.43
Family income (Brazilian minimum wages)		
>2	59	56.19
<2	46	43.81
Economic discontent		
No	18	17.14
Yes	87	82.86
Marital status		
Married	46	43.81
Unmarried	59	56.19
Children		
0	29	27.62
1-2	70	66.67
3-4	06	5.71
Employment relationship		
Civil servant	100	95.20
Contract employee	5	4.80
Ever experienced violence at the FHU		
No	38	36.19
Yes	67	63.81

FHU = Family Health Unit.

The sample overall mean stress score was 2.81, which is the cut-off point for low and high levels of occupational stress. A total of 56 participants (53.33%) had a low level of stress and 49 (46.67%) had a high level of stress. The lowest stress score was 1.00 and the highest was 4.61.

The bivariate analysis between sociodemographic variables (predictor variables) and occupational stress (outcome) dichotomized into low and high levels showed that high stress was more prevalent among younger people (51.56%), women (47.92%), people with white (66.67%) and brown skin color (51.16%), among registered nurses (62.50%), dental assistants (62.50%), and certified nurses (56.25%), those with undergraduate/graduate degrees (51.85%), up to 7 years in the FHU (47.37%), not working outside the FHU (48.19%), with a weekly workload of up to 40 hours (48.84%), not working night shifts (49.46%), with an income of up to 2 Brazilian minimum wages (47.83%), happy with their financial position (61.11%), married (47.83%), with 1 to 2 children (55.71%), a permanent job (49%), and those who had already experienced some violence at the FHU (55.22%), as shown in Table 2. Only the analyses of stress with the variables children, employment relationship, and violence at work were statistically significant, with p-values of 0.01, 0.032, and 0.02, respectively.

**Table 2:** Bivariate analysis between the sociodemographic independent variables and the outcome variable occupational stress

Characteristics	Low stress (n = 56)		High stress (n = 49)		p-value
	n	%	n	%	
Age (years)					
<45	31	48,44	33	51,56	0,210
>45	25	60,98	16	39,02	
Sex					0,407
Male	6	66,67	03	33,33	
Female	50	52,08	46	47,92	
Skin color					0,220
White	3	33,33	6	66,67	
Brown	21	48,84	22	51,16	
Black	32	60,38	21	39,62	
Professional title					0,358
Physician	6	85,71	1	14,29	
Registered nurse	3	3750	5	62,50	
Dentist	5	62,50	3	37,50	
Dental assistant	3	3750	5	62,50	
Certified nurse	7	43,75	9	56,25	
Health community agent	32	55,17	26	44,83	
Education					0,273
Undergraduate/graduate	26	48,15	28	51,85	
Elementary/high school	30	58,82	21	41,18	
Occupation time at the FHU (years)					0,875
<7	30	52,63	27	47,37	
>7	26	54,17	22	45,83	
Works outside the FHU					0,543
No	43	51,81	40	48,19	
Yes	13	59,09	9	40,91	
Weekly working hours					0,346
<40	44	51,16	42	48,84	
>40	12	63,16	7	36,84	
Night shirt					0,123
No	47	50,54	46	49,46	
Yes	9	75,00	3	25,00	
Family income (Brazilian minimum wages)					0,833
>2	32	52,24	27	45,76	
<2	24	52,17	22	4783	
Economic discontent					0,177
No	7	38,89	11	61,11	
Yes	49	56,32	38	43,68	
Marital status					0,833
Married	24	52,17	22	47,83	
Unmarried	32	54,24	27	45,76	
Children					0,01*
0	19	65,52	10	34,48	
1-2	31	44,19	39	55,71	
3-4	6	100,00	-	-	
Employment relationship					0,032*
Civil servant	51	51,00	49	49,00	
Contract employee	5	100,00	-	-	
Ever experienced violence at the FHU					0,02*
No	26	68,42	12	31,58	
Yes	30	44,78	37	55,22	

*p < 0.05 statistically significant.

FHU = Family Health Unit.

Among the items that make up the WSS, those with the highest means were, on a decreasing scale: I have been annoyed with poor training for professional qualification (4.03); I have been distressed by limited opportunities for career growth (3.90); I have been annoyed with poor disclosure of information about organizational decisions (3.62); I have been annoyed with discrimination/favoritism in my workplace (3.29); I have been stressed with the lack of autonomy in performing my duties (3.13), represented in Chart 1.

**Chart 1:** Means and standard deviations of the Work Stress Scale (WSS) items

Statement	Mean	Standard deviation
I have been nervous about the way tasks are distributed in my workplace	3.08	1.144504
The sort of control that exists in my job irritates me	2.92	0997065
Not enough autonomy in carrying out my work has been stressful	3.13	1.000641
I have been annoyed with my manager’s lack of confidence in my work	2.38	1.219875
I have been annoyed with poor disclosure of information about organizational decisions	3.62	1.227732
I have felt annoyed with not enough feedback about my tasks at work	2.88	1.154542
Failing communication between me and my coworkers makes me irritated	2.84	1.093018
I have felt annoyed that my manager has treated me badly in front of my coworkers	2.14	1.28922
I have felt uncomfortable having to do tasks that are beyond my capacity	2.88	1.198519
I have gotten in a bad mood because I have to work for so many hours at a time	2.49	1.119098
I have felt uncomfortable with the communication between me and my manager	2.26	1.074428
I have been annoyed with discrimination/favoritism in my workplace	3.29	1.270401
I have been annoyed with poor training for professional qualification	4.03	1.087138
I have gotten in a bad mood because I feel isolated in the organization	2.36	1.093018
I have become irritated at being undervalued by my managers	2.90	1.304753
I have been distressed by the limited opportunities for career growth	3.90	1.172799
I have been uncomfortable working on tasks below my skill level	2.55	1.100533
Competition in my workplace has put me in a bad mood	2.52	1.066043
Not understanding what my responsibilities are in this job has caused me irritation	2.94	1.15049
I have been nervous about my manager giving me contradictory instructions	2.43	1.116805
I have felt irritated when my manager conceals my good work from other people	2.24	1.014546
Not enough time to do my workload makes has made me nervous	2.56	1.055424
I have been upset that my manager avoids assigning me important responsibilities	2.28	1.004751

## DISCUSSION

Studies covering the mental health of FHS health care personnel are scarce, especially during the COVID-19 pandemic. Currently most studies deal with nursing and specialized health care personnel, leaving gaps in the state of the art when it comes to other professional groups and Primary Health Care, such as the FHS.

The frequency of women in this study is consistent with the increasing number of women in professional roles in the FHS observed in the literature.^14,15,16^ The prevalence of high stress in this group was of 47.92%, leading to the assumption that double or even triple shifts often experienced by women may contribute to this rate.^17^

The highest prevalence of high stress was among workers up to 45 years old (51.56%). Professional stress in younger people can be analyzed based on their reduced work experience and, therefore, less ability to master/control situations in the workplace.^18,19^ This same reasoning can be applied to those who have worked at the FHU for up to 7 years (47.37% were highly stressed).

White women had a higher level of stress (66.67%). Contrary to this result, a study showed a positive and significant association between mental disorders resulting from occupational stressors and black women.^20^

Nursing professionals, namely registered nurses and certified nurses, had a high prevalence of high-level stress. These workers accumulate demands and responsibilities related to providing care for the population, as well as an inadequate physical structure and insufficient materials.^21^ It should be noted that the health units were chosen for vaccination and/or COVID-19 testing, resulting in longer working hours and increased workload.

Workers with an undergraduate/graduate degree had a higher prevalence of high stress, suggesting that the accumulation of responsibilities may be an important stress factor.

The Política Nacional de Atenção Básica (Brazil’s National Primary Care Policy, PNAB) set 40 working hours weekly for workers in family health teams. However, in order to supplement their income, some professionals have other employment relationships, which increases their working hours and workload. However, the categories not working outside the FHU, working up to 40 hours a week, and not working night shifts showed higher frequencies of high stress (48.19%, 48.84%, and 49.46%, respectively) when compared to working outside the FHU, working more than 40 hours a week, and working night shifts, with no statistically significant relationship. No association was found between time at the institution and working shifts and occupational stress.^18^ These professionals’ double shifts possibly lead them to resort to coping strategies and resistance to occupational stressors.

Among those who had an income of up to 2 minimum wages, 47.83% had high stress. Low pay is a trigger for occupational stress^21^ and may be linked to feelings of devaluation and a reduction in purchasing power. However, among those who were happy with their economic position, 61.11% had high stress, compared to 43.68% among those who were economic discontent. This analysis was not statistically significant.

The bivariate analysis found statistical significance in the relationship between the variables children, work relationship, and violence at the FHU.

Childcare can be a stressor that adds to a person’s routine, especially when represented by women, who are generally responsible for childcare.^19^ In contrast, a study concluded that having children was a protective factor against burnout syndrome in professionals at a hospital in Spain.^22^

Employment stability is considered a protective factor against stress. However, a finding contradicted these results, where the level of moderate stress was very close between both civil servants and outsourced workers.^23^ The 5 professionals working as contracted workers are physicians who, despite their temporary employment, earn higher salaries than the other categories, which may mitigate stress.

Most participants reported having experienced some kind of violence in the workplace (63.81%); 55.22% had a high level of stress (p = 0.02), with reports of verbal aggression, psychological violence, moral harassment, and even cases of physical aggression, thus triggering occupational stress.^24,25^

Violence and abuse against nursing personnel had been reported in several countries even before the pandemic. Poor working conditions, which have an impact on the provision of care to the population, are the main causes of violence. During the COVID-19 pandemic, institutional violence against nursing personnel has increased, in addition to discrimination against health care personnel as they provide frontline care to patients. Violence characteristics remained similar to the pre-pandemic period, such as verbal and physical aggression and moral harassment.^26^

Although health personnel were considered heroes because of their work during the pandemic, the acts of violence against them seem to contradict both the population that needs their care and the managers, who do not provide satisfactory environmental, structural, or material conditions, nor the proper financial appreciation for these workers.^26,27^

A study with nursing personnel in intensive care units concluded that 70.8% of the participants had moderate stress and 18.1% had high stress.^28^ A study conducted during the COVID-19 pandemic found a high prevalence of psychological distress, perceived stress, and burnout syndrome among frontline health care personnel.^29^

Mental distress had a prevalence of 61.6% among health care personnel from different categories and all levels of health care working during the pandemic. Individual factors such as age under 40 and being a woman were associated with mental distress, as were psychosocial factors such as working more than 60 hours a week and low support from coworkers.^30^

On the other hand, a nursing team working at hospitals showed that 53.8% had anxiety, 38.4% had depression, and 40.3% had stress, which was associated with length of service, employment contract, and job satisfaction.^31^ Another study with nursing personnel found that 90.6% had occupational stress, which was associated with a higher level of education, income, and care.^32^

Most studies are aimed at nursing personnel and specialized health care.^33^ Our study, on the other hand, looks at Primary Health Care and includes all health care personnel working with FHS.

According to the literature, the pandemic has contributed to exacerbating occupational stress among health care personnel due to longer working hours, increased workload, insufficient resources and protective equipment, fear of infection, loss of patients and relatives, among other factors.^29,30,33^

The lowest stress score was 1.00 and the highest was 4.61. In the analysis of occupational stress in residents of a multi-professional program, stress scores ranged from 1.04 to 4.39 for those in the first year of the program and from 1.61 to 4.65 for those in the second year of the program.^34^

Among the WSS items, the one that stood out with the highest mean was poor training for professional qualification (4.03), followed by limited opportunities for career growth (3.90); poor disclosure of information about organizational decisions (3.62); discrimination/favoritism in the workplace (3.29) and lack of autonomy in performing duties (3.13). Another study, conducted before the pandemic, found that the main stressors were a lack of information on organizational decision-making (2.97), poor career growth opportunities (2.75), limited training and professional qualification (2.69), irritation due to control in the workplace (2.59), and being nervous about the way tasks were distributed at the workplace (2.58).^23^ When compared to the study conducted before the pandemic, the most stressful factors were similar in three items. To date, no studies have used the WSS questionnaire with health care personnel during the COVID-19 pandemic.

Thus, the highest mean scores found in the WSS items can guide local management to take actions that address the real needs of health care personnel, such as permanent qualification, financial appreciation of workers through compliance with the job and salary plan, access to organizational information for all, improvements in interpersonal relations, and more participation of workers in decision-making in the workplace.^13^

This study was conducted in only one HD in Salvador, Brazil. This is therefore a limitation of the study, given that data were collected from a geographically bounded demographic group.

Cross-sectional studies have the disadvantage of not measuring risks or causal relationships. On the other hand, it allows prevalence and prevalence ratios to be measured.^11^ However, this study was limited to a descriptive analysis of the participants sociodemographic and work-related variables, and the prevalence of high levels of stress among independent variables using bivariate analysis, which could contribute to occupational health planning aimed at mitigating occupational stress among FHS health care personnel.

## CONCLUSIONS

High levels of occupational stress were highly prevalent (46.7%) among health care personnel at FHUs during the COVID-19 pandemic, with statistical significance in the bivariate analysis with the variables children (p = 0.01), employment relationship (p = 0.032), and violence at workplace (p = 0.02).

Among the main stressors identified using WSS are poor professional training, few opportunities for career growth, poor disclosure of organizational information, discrimination and favoritism in the workplace, and not enough autonomy to perform their duties, which could guide managers in planning actions to address these demands.

Further studies could be conducted on the mental health of health care personnel, covering other professional groups and Primary Health Care.
